# Maximising public adherence to COVID-19 self-isolation in Europe

**DOI:** 10.1016/j.lanepe.2021.100089

**Published:** 2021-03-21

**Authors:** Jay Patel, Genevie Fernandes, Devi Sridhar

**Affiliations:** a Faculty of Medicine and Health, University of Leeds, Worsley Building, Clarendon Way, Leeds LS2 9JT, UK; b Global Health Governance Programme, Usher Institute, University of Edinburgh, Edinburgh, UK

Despite the marked international variance in COVID-19 related public health strategies, several fundamentals remain critical for controlling the pandemic across all nations. Crucially, the management of suspected and confirmed cases and their close contacts is a key determinant of infection rates in a population [1].Therefore, an efficiently-operating test-trace-isolate system is of paramount importance to interrupt community transmission, with particular emphasis on providing support for isolation for a specified period (usually between 10 and 14 days across Europe). Drawing on analyses of international approaches to self-isolation measures [2], we distil five key insights for maximising public adherence to self-isolation in the European region.

First, without sufficient support, self-isolation is an infeasible proposition for many. Comprehensive support, prioritising financial and practical support strands should be provided to encourage and enable self-isolation. While several European countries offer statutory sick pay and some provide additional allowance, commensurate and accessible financial support should be offered to anyone who has to self-isolate regardless of eligibility criteria. Throughout the pandemic, evidence has shown that disincentives––without support––are unlikely to coax individuals to comply with voluntary guidelines [3]. In the Czech Republic, which has recorded one of the highest proportion of deaths per million worldwide (1940) [4], the lack of meaningful financial support to enable adherence to self-isolation instructions is often cited as a driver for this outcome. Those required to isolate, are entitled to 60% of their salary for the first two weeks, which many citizens consider insufficient.

As with the majority of health protective behaviours, public willingness to comply is generally high, but often the practical feasibility has prevented full adherence [5]. Cities in Belgium have set up a municipal website where volunteers who want to help and people who need help can register to find each other [2]. More of such initiatives linking the public with local health teams and community based organisations and volunteer networks can enable the provision of a range of practical services including food supplies, caregiving and mental health support.

Second, the provision of alternative temporary accommodation should be considered to allow the safe physical distancing of household members, especially in crowded or intergenerational settings. Indoor settings are notably associated with higher risk of transmission, and attributed with a 16% secondary attack rate [6]. Greater reduction in transmission is possible when people are able to isolate outside their household [1]. Subject to eligibility criteria, some countries (for example, Denmark, Finland, France, Italy, the Netherlands, Norway, and Slovakia) offer accommodation for the duration of the stipulated isolation period, or reimburse costs required to stay at a hotel or other quarantine facility. New York City have managed quarantine facilities, which cater for individual support needs, whilst in a safe environment. Government supported partnerships with hotel associations could be set up to enable safe self-isolation and support local economies.

Third, clear and accessible public health messaging, communicating when and how to self-isolate is warranted. Rapid isolation during the infectious period, soon after symptom onset or receipt of test result, confers significant benefit in reducing transmission. It is plausible that some individuals are only isolating on the basis of a positive test result or on becoming symptomatic, as studies in the Netherlands [7], and Norway have shown [8]. Low levels of awareness on government guidance, particularly regarding what to do upon developing symptoms was strongly associated with non-adherence to self-isolation in the UK [9]. As the rationale for self-isolation is relevant in predicting compliance, there is a clear need for targeted informational support, encouraging adherence on a precautionary basis, especially whilst awaiting a test result.

Fourth, test-trace-isolate systems need to be strengthened under decentralised coordination. Effective contact tracing is dependent on trust, which holds greater value when conducted by local public health teams and in countries with high levels of political trust [10]. In a number of European countries, public health authorities are already implementing testing and contact tracing at regional and local levels; linking support measures to these local test-trace-isolate systems will be beneficial in improving adherence to protective behaviours.

Finally, routinely monitoring actual adherence to self-isolation and collecting information on barriers and facilitators is required to understand how test-trace-isolate systems work and how they can be improved. The Netherlands offers a strong example, where the National Institute of Public Health and Municipal Public Health Service conduct regular household surveys capturing COVID-19 behaviours [7]. Involving primary care workers, local community health workers and volunteers to collect such information in a safe and secure manner could encourage individual to report their actual behaviours as well as concerns.

COVID-19 has exacerbated inequalities and there are individuals and whole communities that cannot isolate without adequate financial and practical support that is easily accessible. We must accept this reality and unequivocally push for measures that support this critical behaviour.

## Author Contributions

JP and GF jointly collated data and drafted the manuscript. DS critically revised the draft. All authors conceived this commentary and approved the final version of the manuscript.

## Financial support

We acknowledge support of Wellcome Trust [106635/Z/14/Z].

## Declaration of Interests

DS is on the Scottish Government COVID-19 advisory group, on the Royal Society DELVE group that feeds into SAGE and a member of the UK Cabinet Office's International Joint Comparisons Unit. JP and GF have no conflicts of interest to declare.
